# Supplementary material for: Can a Mixture of Farnesene Isomers Avert the Infestation of Aphids in Sugar Beet Crops?
Source: Insects. 2024 Sep 24;15(10):736. doi: 10.3390/insects15100736 (PMC11508235; doi:10.3390/insects15100736)
Supplement: Supplementary file 1 [file insects-15-00736-s001.zip › insects-3207384-supplementary.pdf]

Supplementary material

Can a mixture of farnesene isomers avert the infestation of aphids in sugar beet crops?

Denise Kuhn [1,2], Nils Nägele [2], Till Tolasch [1], Georg Petschenka [2] and Johannes L.M. Steidle [1,*]

[1] Department of Chemical Ecology 190t, Institute of Biology, University of Hohenheim, 70599 Stuttgart, Germany, denise.kuhn@uni-hohenheim.de
[2] Department of Applied Entomology 360c, Institute of Phytomedicine, University of Hohenheim, 70599 Stuttgart, Germany
* Correspondence: author: Johannes.steidle@uni-hohenheim.de

Table S1. Total number of natural enemies found on the sugar beet plants.

Day after application/ Location	Treatment	0	7	14	21	28	35	42
Mannheim	Control	0	0	1	5	0	0	3
	FIM	0	0	1	3	1	9	2
Heidelberg	Control	1	0	5	21	5	4	3
	FIM	0	5	1	21	9	5	3
Dettenheim	Control	0	1	0	3	3	12	4
	FIM	0	0	0	7	3	7	4
